# Personal Experience of *Daboia siamensis* Envenomation

**DOI:** 10.1155/2021/3396373

**Published:** 2021-12-24

**Authors:** Tein-Shun Tsai, Chun-Chieh Liu, Po-Chun Chuang

**Affiliations:** ^1^Department of Biological Science and Technology, National Pingtung University of Science and Technology, Pingtung, Taiwan; ^2^Department of Emergency Medicine, Ministry of Health and Welfare Pingtung Hospital, Pingtung, Taiwan; ^3^Department of Emergency Medicine, Kaohsiung Chang Gung Memorial Hospital, Kaohsiung, Taiwan

## Abstract

Reports of envenomation induced by *Daboia siamensis*, a medically important venomous snake in Taiwan, are rare, and species identification might not be definitive. This article reports the complete course of a definite *D. siamensis* bite. The patient in this report was one of the authors who was bitten on the right palm near the base of the index finger by *D. siamensis.* The patient experienced local effects, neurological manifestations, and acute kidney injury. The laboratory analysis revealed elevated D-dimer and coagulopathy. The patient was administered 8 vials of antivenom and did not undergo surgical intervention or endotracheal tube intubation, but serum sickness occurred 8 days after antivenom administration. The horse immunoglobulin produced by the Centers for Disease Control, R. O. C. (Taiwan), against *D. siamensis* was effective and safe in the treatment of the patient. However, the best antivenom administration strategy remains unclear and requires further study.

## 1. Introduction

Patients who experience the bite of *Daboia siamensis*, a medically important venomous snake in Taiwan, are mainly resident in the southern and eastern areas of Taiwan [1] The major symptoms of *D. siamensis* bites in envenomed patients include local effects, varying degrees of acute kidney injury, incoagulable blood with bleeding tendency, and hemolysis [2, 3]. Before the availability of a specific antivenom in Taiwan in August 2008, the bite of *D. siamensis* species induced more serious kidney injury than that of other Russell's vipers in Southeast Asia [4–6]. However, reports on the treatment of patients bitten by *D. siamensis* remain rare, and species identification might not be definitive. This article reports the complete course of a definite *D. siamensis* bite in a patient.

## 2. Case Report

**Figure 1:**
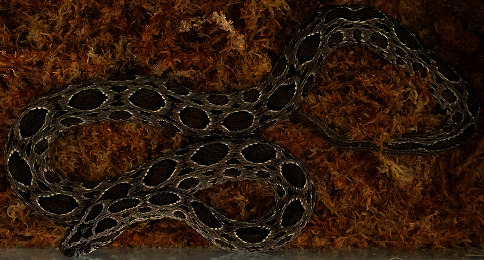
The culprit *D. siamensis*. A male *D. siamensis* originally captured from Kaohsiung City and housed in a reptile and amphibian facility; snout-vent length = 66.7 cm, tail length = 12.4 cm, bodyweight = 220 g.

One of the authors (TS Tsai, a 48-year-old man) was bitten on the right palm near the base of the index finger by *D. siamensis* (Figure 1) in the Reptile and Amphibian Facility at the National Pingtung University of Science and Technology in Pingtung, Taiwan. The accident might have occurred when the patient's fingers stuck to the skin of the snake when he grasped the neck of the snake to inspect its mouth and nostrils for symptoms and signs of infection. After inspection, the patient attempted to release the snake back into the snake box, and it then turned its head around and bit the patient's hand while he was releasing it. Two fang bite marks, scratches, and bruises were visible on the spot.

**Figure 2:**
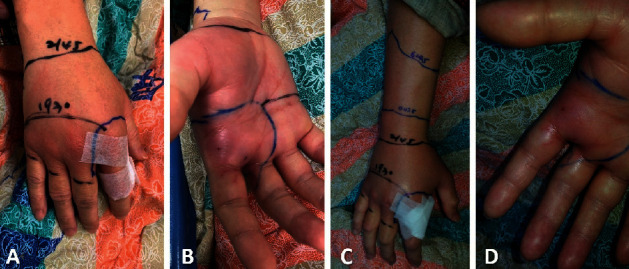
Wounds of the *D. siamensis* bite. Wound on ((a)-(b)) day 1 and ((c)-(d)) day 2. (b), (d) Two fang bite marks and some scratches or bruises visible.

The patient started to feel numbness and tingling of the ankles and experienced slight difficulty in walking approximately 30 min after the snakebite. The patient visited a local hospital 30 min later and was administered 4 vials of monovalent antivenom for *D. siamensis* at the emergency department within 1 h of being bitten. However, the swelling and redness had progressed to the wrist (Figure 2) approximately 9 h after the snakebite. Therefore, 4 more vials of antivenom were administered, for a total of 8 vials within 10 h. Blood tests showed acute kidney injury, disseminated intravascular coagulation, and coagulopathy (Table 1). No leukocytosis or anemia was observed, and antibiotics, such as ceftazidime, were prescribed.

The patient was admitted after which the right hand showed the most obvious swelling and pain for the first 2-3 days. The swelling and pain even extended to the right elbow. The patients' fingers were almost unable to bend. Ice pillow packing was used to relieve the pain, local heat, and swelling. Fortunately, there were no blisters, hemorrhagic bullae, or finger ischemia. The patient also received two courses of hyperbaric oxygen treatment on days 3 and 4, and the swelling of the hand quickly subsided after that. Then, the patient's fingers were able to bend slightly on the third night and were able to bend more than 90° on day 4. During hospitalization, the patient's daily stool was soft or fluid and dark blue-green but not black, whereas the urine color was normal. The patient was discharged from the hospital on day 5, and on day 8, a large area of elevated red skin rash and itching of the body occurred at home. After administration of antihistamines, the symptoms disappeared. The patient still sometimes experienced mild numbness and painful sensations at the bite site for up to one month after the snake envenomation.

## 3. Discussion

Although early antivenom administration would benefit wound recovery and reduce pain in affected patients [7, 8], the optimal antivenom administration strategy for *D. siamensis* envenomation remains unclear. Compared to a recent study [3], most patients with *D. siamensis* envenomation were administered 4–6 vials of antivenom. The patient in this case was initially administered 4 vials of antivenom. However, the subsequent redness and swelling continued to worsen, extending over the wrist joint, and therefore, another 4 vials were administered. Compared with previous studies, the overall amount of antivenom administered to this patient was more than the average dose, and there were no blisters or hemorrhagic bullae, and surgical intervention was not required [3].

The weak neurotoxic manifestations observed in this patient were numbness and pain at the ankles, general weakness, and difficulty in walking at the initial stage after the snakebite. Similar to previous studies, ptosis or respiratory failure did not occur [2, 3]. The laboratory data showed elevated D-dimer levels and coagulopathy. Acute kidney injury occurred, but the patient rapidly recovered, which could be due to the early antivenom administration [4]. Although the evidence of the benefits was not clear, ancillary treatments such as antibiotics, ice packing, and hyperbaric oxygen were administered to this patient.

Ceftazidime was used in this case because of the high infection rate and susceptibility to pathogens associated with snakebites in Taiwan [9, 10]. There was no severe antivenom reaction in this case; however, allergies occurred 8 days later. The adverse effect might have serum sickness, which mostly occurs 5–10 days after serum injection. A previous study also reported that administering more antivenom has a higher risk of inducing serum sickness [11].

## 4. Conclusion

This is a complete and definitive report of a case of *D. siamensis* snake bite in Taiwan. The horse immunoglobulin produced by the Centers for Disease Control, ROC (Taiwan), against *D. siamensis* was effective and safe in the treatment of this patient. However, the best antivenom administration strategy remains unclear, and further studies are required to determine an appropriate regimen.

## Figures and Tables

**Table 1:** Laboratory data in time after *Daboia siamensis* snakebites.

	After 1 hour	After 6 hours	After 14 hours	After 38 hours
WBC (1000/*µ*L)	6.7			8.43
Hemoglobin (g/dL)	13.9			12
Platelet (1000/*µ*L)	191	197		179
PT (second)	>50	>50	18.4	12
aPTT (second)	>100	29	25	29
BUN (mg/dL)	20			
Creatinine (mg/dL)	1.05		1.54	1.25
ALT (U/L)	18			
CPK (U/L)	110			
D-dimer (ng/mL)	>10000		>10000	>10000

WBC, white blood cells; PT, prothrombin time; aPTT, activated partial thromboplastin time; ALT, alanine aminotransferase; CPK, creatine phosphokinase.
